# Excess Mortality and Social Vulnerabilities During the 1742–1743 Plague Epidemic: Demographic and Socioeconomic Impacts in Cordova and Santa Fe Along the Royal Road

**DOI:** 10.3390/epidemiologia6010011

**Published:** 2025-03-04

**Authors:** Valentina Villafañe, Jorge Hugo Villafañe

**Affiliations:** 1 Istituto Istruzione Superiore Amaldi Sraffa, Via F.lli Rosselli, 35, 10043 Orbassano, Italy; valentina.villafane45@gmail.com; 2 Departamento de Historia y Filosofía, Universidad de Alcalá, 28801 Alcala de Henares, Spain; 3 Faculty of Medicine, Health and Sports, Universidad Europea de Madrid, 28670 Villaviciosa de Odón, Spain

**Keywords:** epidemic, excess mortality, Latin America

## Abstract

Background/Objectives: The 1742–1743 plague epidemic had a profound impact on populations along the Royal Road (Camino Real), the principal trade route connecting Buenos Aires and Lima. This study aimed to quantify the demographic and socioeconomic consequences of the epidemic in Cordova and Santa Fe, with a focus on excess mortality and its broader implications for marginalized groups. Methods: This research utilized parish death records and complementary historical sources to calculate excess mortality in Cordova and Santa Fe during the epidemic. Mortality rates were compared across pre-epidemic (1740–1741), epidemic (1742–1743), and post-epidemic (1744–1745) periods. Additional data on demographic variables such as age, gender, marital status, and ethnicity were analyzed to identify patterns of vulnerability and resilience. Results: Excess mortality during the epidemic was significant, with death rates in Cordova peaking at 12 times the pre-epidemic average in May 1743, while Santa Fe experienced a 45% increase in mortality, peaking in December 1743. Marginalized groups, including enslaved and Indigenous populations, were disproportionately affected, exacerbating existing social inequalities. The epidemic also disrupted socioeconomic structures and highlighted systemic vulnerabilities in both urban centers. Conclusions: This study demonstrates the critical role of excess mortality as a metric for understanding the demographic and socioeconomic impacts of historical epidemics. By integrating quantitative and qualitative analyses, it underscores the intersection of public health crises with social structures in colonial Latin America. The findings offer insights into resilience and recovery mechanisms relevant to both historical and contemporary public health strategies.

## 1. Introduction

The 1742–1743 plague epidemic represents a significant event in the demographic and social history of colonial South America, with severe consequences for populations along the Royal Road (Camino Real), the primary trade route connecting Buenos Aires and Lima. Although frequently examined in isolation, this epidemic must be contextualized within a broader continuum of outbreaks that afflicted the region over several decades. Epidemiological records trace the origins of the epidemic to the early 18th century, with documented cases emerging in Cuyo as early as 1705. From this focal point, the disease propagated toward the capital, resurfacing at periodic intervals in major urban centers along the Royal Road, including Cordova, Santa Fe, and Buenos Aires. This recurrent transmission underscores the integral role of trade, migration, and colonial administrative networks in the dissemination of infectious diseases within the region.

Urban centers like Cordova and Santa Fe were particularly affected, experiencing notable demographic disruptions and exposing systemic vulnerabilities among marginalized groups, including Indigenous, African-descended, and enslaved populations [1]. The epidemic not only caused substantial mortality but also revealed structural inequities that exacerbated its impact and intensified the challenges faced by vulnerable communities [2].

Although plague is historically associated with devastating pandemics such as the Black Death in Europe, *Yersinia pestis* also has a documented presence in the Americas [3]. The bacterium is believed to have been introduced during the European exploration and colonization period, with outbreaks recorded across various regions of Central and South America between the 16th and 18th centuries. A particularly severe manifestation occurred in 1576 in Mexico-Tenochtitlán, inflicting significant mortality on Indigenous populations. Subsequent outbreaks in Peru, Ecuador, and Brazil coincided with the arrival of European vessels and the expansion of commercial networks, underscoring the pivotal role of trade and mobility in pathogen dissemination [4]. Notably, Yersinia pestis was not endemic to the Americas, but was introduced sporadically, instigating episodic epidemics that disrupted socioeconomic structures [5].

During the 18th century, prevailing medical paradigms were anchored in antiquated humoral theories and empirical methodologies. The predominant belief in the balance of humors informed medical interventions, with clinical observation and traditional therapeutic practices constituting primary responses to disease outbreaks. These historical conceptualizations significantly influenced epidemic management strategies and the resilience demonstrated by affected societies [6].

Epidemics, especially plague, have historically reshaped societies, as widely documented in medieval Europe [4]. However, the effects of 18th-century outbreaks in South America remain underexplored, particularly regarding their demographic toll and broader social implications [7]. This study addresses this historiographical lacuna by utilizing excess mortality as a critical metric to assess the epidemic’s impact and examine its influence on population dynamics and socioeconomic structures along the Royal Road. Unlike European plague outbreaks, often exacerbated by global trade and climatic fluctuations, the dissemination of this epidemic was likely intensified by regional mobility patterns and interdependent colonial networks, amplifying extant social and economic disparities [8,9].

Archival sources indicate that the epidemic devastated cities such as Cordova, Santa Fe, and Buenos Aires, engendering extensive socioeconomic disruption. This investigation leveraged parish death records, population censuses, and complementary archival materials to delineate the epidemic’s demographic consequences, economic perturbations, and patterns of community resilience [10]. While previous localized studies on Cordova and Buenos Aires have yielded valuable insights, regional epidemic dynamics, particularly in interconnected nodes like Santa Fe, remain insufficiently scrutinized. Furthermore, Jesuit records from the Chiquitos missions suggest that the epidemic disseminated along trade and missionary routes, revealing systemic vulnerabilities within colonial infrastructure [11,12,13].

Adopting a multidimensional approach, this study investigated the demographic, social, and economic impacts of the 1742–1743 plague epidemic, contextualizing it within the broader epidemiological landscape of 18th-century South America. A comparative analysis of Cordova and Santa Fe elucidates how their strategic positions along the Royal Road influenced epidemic exposure, mortality patterns, and post-crisis recovery trajectories. These inter-regional comparisons yield critical insights into the interplay between local and structural determinants in shaping epidemic outcomes and societal responses.

A comprehensive analysis of this epidemic not only broadens our understanding of historical health crises but also establishes a framework for examining the interplay between disease, population shifts, and socioeconomic structures in colonial Latin America. This perspective underscores the interconnected nature of local and regional factors that shaped the epidemic’s outcomes, offering valuable lessons for both historical and contemporary public health strategies [14].

This research integrated parish death records, population censuses, and other archival materials to quantify the excess mortality caused by the epidemic and to assess its broader socioeconomic repercussions. The objective of this study was to calculate the excess mortality associated with the epidemic and explore its implications for population dynamics and social structures, particularly among marginalized groups along the Royal Road. By focusing on the demographic toll and the resilience demonstrated by local communities amid significant mortality and socioeconomic upheaval, this study contributes to a deeper understanding of the long-term impacts of health crises on colonial societies in Latin America.

## 2. Materials and Methods

This study evaluated the demographic impact of the 1742–1743 plague epidemic in the cities of Cordova and Santa Fe, two key locations along the Royal Road, the primary trade route connecting Buenos Aires and Lima. Excess mortality was used as the main metric, allowing for the quantification of both direct deaths caused by the disease and indirect deaths related to the social and healthcare disruptions triggered by the epidemic.

### 2.1. Estimation of Excess Mortality

Excess mortality estimation relied on parish records from both cities, selected for their consistency and reliability. Death counts during the epidemic were compared with those from the pre- and post-epidemic periods, defined as follows [11,15]:

- Pre-epidemic period: January 1740–December 1741.
- Epidemic period: January 1742–December 1743.
- Post-epidemic period: January 1744–December 1745.

This comparison enabled the precise quantification of deaths attributable to the epidemic, highlighting its effects on urban centers of varying complexity.

### 2.2. Review of Primary Sources

Parish death registers were complemented with local census data, baptismal and marriage records, and colonial administrative documents to address potential data gaps, such as missing information on age or marital status. The 1744 census in particular provided critical insights into demographic recovery following the epidemic.

### 2.3. Definitions and Data Access

Marginalized social groups were identified based on socioeconomic stratification, occupational categorization, and patterns of residential segregation as documented in historical census records and parish archives. These criteria were formulated in alignment with historiographical standards and prior demographic studies [11,15,16].

The archival records were manually retrieved from parish archives and historical repositories. Access to these sources was granted through official archival institutions, and data extraction adhered to standardized procedures designed to ensure the fidelity and comparability of historical records.

### 2.4. Considerations on the Record-Keeping System

Mortality data from Cordova and Santa Fe were systematically analyzed to identify convergences and regional variations, emphasizing the role of each city’s strategic location along the Royal Road in shaping its epidemic trajectory. A demographic and socioeconomic analysis was conducted, integrating variables such as sex, age, marital status, and ethnicity to elucidate differential mortality patterns.

### 2.5. Methodological Adjustments for Historical Limitations

Given the diagnostic constraints of 18th-century record-keeping, methodological adjustments were applied to account for possible underreporting of deaths. This included harmonizing data between cities and benchmarking against contemporary metrics of excess mortality to ensure the robustness of the analysis.

The analysis also considered external variables influencing mortality trends, such as:

- Climatic factors: temperature, humidity, altitude, and extreme weather events.
- Time–distance variables: proximity between settlements and socioeconomic hierarchies.
- Trade networks: their potential role in the propagation of the epidemic.

This methodological framework provides a comprehensive understanding of the demographic and social impact of the epidemic in Cordova and Santa Fe, highlighting the interactions among mortality, social structures, and economic dynamics along the Royal Road.

### 2.6. Data Analysis

Statistical analyses were performed using SPSS version 28.0 to assess biodemographic patterns, including estimations of consanguinity levels and demographic shifts. Geographic information systems (GISs) were employed for spatial analysis, facilitating territorial adjustments and the delineation of affected areas.

Excess mortality estimates were derived through validated demographic methodologies applied to historical datasets. Adjustments were made via four principal operations:

- Territorial adjustment.
- Chronological adjustment.
- Socioeconomic stratification analysis.
- Urbanization and settlement type classification.

Furthermore, cross-referencing with census records enabled the verification of demographic conditions and mortality trends, reinforcing the validity of the analytical approach.

## 3. Results

### 3.1. Epidemiological Characteristics of the Plague in Cordova and Santa Fe

#### 3.1.1. Comparative Mortality

Table 1 summarizes the mortality data for Cordova and Santa Fe across three phases: pre-epidemic, epidemic, and post-epidemic. A significant increase in mortality was observed during the epidemic phase (1742–1743), with Cordova experiencing a 3.1-fold rise compared to the pre-epidemic period, while Santa Fe recorded a 45% increase. Mortality in Cordova peaked in May 1743, whereas Santa Fe’s peak occurred later, in December 1743.

#### 3.1.2. Mortality by Sex, Age, and Marital Status

The demographic breakdown of mortality in Table 2 reveals important patterns during the epidemic phase. Both Cordova and Santa Fe experienced a notable rise in adult mortality (individuals ≥ 18 years), which increased from 62.2% in the pre-epidemic period to 76.8% during the outbreak. Female mortality showed a slight increase in Cordova (from 47.3% to 48.1%), whereas Santa Fe exhibited a marginal decline. Notably, the proportion of single individuals among the deceased dropped considerably during the epidemic.

#### 3.1.3. Ethnic Composition

The ethnic distribution of mortality highlights the disproportionate impact of the plague on marginalized populations, including Indigenous, African descent, and *pardo* groups (Table 3). Both Cordova and Santa Fe reflected pre-existing social and ethnic inequalities during the epidemic. Creole populations experienced a relative increase in mortality, while Indigenous and African-descent groups, although severely affected, showed a slight decline compared to the pre-epidemic phase. The *pardo* and mestizo groups exhibited a notable reduction during and after the epidemic.

## 4. Discussion

This study provides an analysis of the demographic and socioeconomic impacts of the 1742–1743 plague epidemic in Cordova and Santa Fe, two of the principal cities along the Royal Road. By focusing on these strategic urban centers, the study highlights the epidemic’s magnitude, its disproportionate effects on marginalized groups, and the systemic vulnerabilities it exposed within the interconnected trade networks of colonial South America. This localized approach allows for a deeper understanding of how the epidemic’s dynamics unfolded within these pivotal nodes of the Royal Road.

### 4.1. Excess Mortality and Vulnerable Populations

The epidemic’s demographic toll varied significantly between Cordova and Santa Fe, illustrating both shared vulnerabilities and distinct local dynamics. In Cordova, mortality increased up to 3.1 times pre-epidemic levels, peaking at 12 times in May 1743, while Santa Fe saw a 45% rise in mortality, with the highest number of deaths recorded in December 1743. Marginalized groups bore the brunt of these increases: enslaved individuals and Indigenous populations in Cordova accounted for over 60% of deaths during the epidemic, while in Santa Fe, the *pardo* population—often at the intersection of African and Indigenous identities—experienced a dramatic increase in mortality, rising from 7.4% pre-epidemic to 23.4% during the outbreak.

These demographic patterns align with broader structural inequalities documented in colonial contexts, where systemic inequities exacerbated the vulnerability of marginalized populations during epidemics [2,12].

### 4.2. Demographic and Socioeconomic Disruptions

The epidemic not only increased mortality but also disrupted labor forces and social structures in both cities. In Cordova, adults (≥18 years) comprised 76.8% of deaths during the epidemic compared to 62.2% pre-epidemic, while Santa Fe showed a similar trend, with adults accounting for 69.5% of deaths during the epidemic, up from 57.4% pre-epidemic. These shifts underscore the epidemic’s destabilizing effect on local economies, particularly through the loss of working-age individuals.

Ethnic disparities further illustrate the epidemic’s socioeconomic toll. In Cordova, Indigenous and African-descended populations experienced the highest mortality rates, highlighting entrenched inequities. Santa Fe exhibited comparable patterns, with marginalized groups disproportionately affected. These findings resonate with historiographical discussions on the interplay between epidemics and socioeconomic structures in colonial Latin America, demonstrating how health crises often amplify existing inequalities [1].

### 4.3. The Role of the Royal Road in Epidemic Dynamics

The Royal Road functioned as both a conduit for economic growth and a vector for epidemic spread. The movement of goods and people facilitated the rapid transmission of the plague, linking localized outbreaks to broader disruptions. Historical records emphasize how interconnected trade and migration networks intensified vulnerabilities to health crises [13,17].

Environmental factors, including deforestation and climatic variability, likely influenced the dynamics of plague reservoirs and vectors. The interaction between these environmental conditions and socioeconomic activities created feedback loops that exacerbated the epidemic’s impact on vulnerable populations. Understanding this interplay is essential for comprehensive epidemic analyses [18,19].

### 4.4. Community Resilience and Recovery

Despite the epidemic’s devastating impacts, both Cordova and Santa Fe demonstrated remarkable resilience during the recovery period. Religious and cultural practices, such as novenas and public prayers, played crucial roles in re-establishing social cohesion. In Santa Fe, novenas dedicated to Saints Jerónimo and Roque addressed both the epidemic and concurrent drought conditions, illustrating the integration of spiritual and administrative responses to public health crises. These practices were complemented by adaptive strategies, such as the diversification of production units in Cordova and localized recovery measures in Santa Fe, which highlight the communities’ capacity to rebuild social and economic structures.

These responses exemplify how resilience emerged from a combination of community-driven initiatives and institutional measures. Efforts by colonial authorities, such as quarantines and administrative reforms, provided critical support for recovery and long-term social reorganization [2].

### 4.5. Comparative Patterns with the COVID-19 Pandemic

The parallels between the 1742–1743 plague epidemic and the COVID-19 pandemic are notable, particularly in their demographic and socioeconomic impacts. During the 2020–2021 biennium, Cordova and Santa Fe experienced significant increases in mortality due to COVID-19. For instance, Santa Fe reported 310,805 confirmed cases (approximately 89 cases per 1000 inhabitants) and 4948 deaths, while Cordova recorded an 8.6% increase in excess mortality. Both crises disproportionately affected marginalized and vulnerable populations, exacerbating pre-existing inequalities and exposing the fragility of health systems [10,20].

These comparisons reveal the cyclical nature of epidemics, where periods of acute crisis are followed by stabilization phases with lasting demographic and socioeconomic effects [21]. Both historical and contemporary cases underscore the importance of addressing systemic vulnerabilities to mitigate the impact of health crises. By synthesizing historical and modern data, this study highlights the relevance of equity-focused strategies to strengthen resilience and recovery frameworks.

### 4.6. Limitations of the Study

This study is subject to several limitations inherent to its historical and methodological framework. The reliance on parish death records introduces potential biases due to incomplete or inconsistent documentation. Cause-of-death records in the 18th century often reflected cultural or religious interpretations rather than medical diagnoses, complicating the attribution of mortality to the plague. Additionally, underreporting and the absence of standardized record-keeping pose further challenges to precise mortality estimations.

The use of excess mortality as a key metric assumes stable demographic trends during the pre- and post-epidemic periods. However, factors such as migration, natural disasters, or other concurrent diseases may have influenced mortality rates, potentially leading to over- or underestimation of the epidemic’s impact.

Furthermore, the study’s analysis of Cordova and Santa Fe, while offering valuable insights, may not fully capture the diverse regional variations in epidemic dynamics along the Royal Road. Factors such as environmental conditions, socioeconomic structures, and local responses could have varied significantly across other settlements.

Lastly, while this study integrates socioeconomic and demographic perspectives, it does not extensively incorporate environmental or biological analyses, such as the role of plague reservoirs and vectors. Future research could benefit from a more interdisciplinary approach, combining historical, biological, and environmental data to deepen our understanding of epidemic spread and its impacts in colonial Latin America.

## 5. Conclusions

The 1742–1743 plague epidemic profoundly affected Cordova and Santa Fe, revealing the vulnerabilities of interconnected trade networks and marginalized populations. Excess mortality, as a central metric, highlights the epidemic’s disproportionate toll and its broader socioeconomic consequences. By examining these historical patterns, this study contributes to a deeper understanding of the resilience mechanisms that enabled recovery, emphasizing the importance of equity-driven and culturally informed public health strategies for addressing future health crises.

## Figures and Tables

**Table 1:** Comparative mortality rates during the pre-epidemic, epidemic, and post-epidemic phases of the 1742–1743 plague in the city of Cordova and Santa Fe.

Month	Pre-Plague, 1740–1741	Plague, 1742–1743	Post-Plague, 1744–1745
January	17 (7.0%)	54 (9.7%)	21 (7.8%)
February	16 (6.6%)	28 (5.1%)	19 (7.1%)
March	11 (4.5%)	32 (5.8%)	20 (7.4%)
April	24 (9.9%)	59 (10.6%)	22 (8.2%)
May	10 (4.1%)	52 (9.4%)	22 (8.2%)
June	17 (7.0%)	46 (8.3%)	25 (9.3%)
July	19 (7.8%)	37 (6.7%)	26 (9.7%)
August	21 (8.6%)	49 (8.8%)	24 (8.9%)
September	15 (6.2%)	47 (8.5%)	23 (8.6%)
October	19 (7.8%)	50 (9.0%)	25 (9.3%)
November	34 (14.0%)	49 (8.8%)	15 (5.6%)
December	40 (16.5%)	51 (9.2%)	27 (10.0%)
Total	243 (100%)	554 (100%)	269 (100%)

**Table 2:** Mortality distribution by sex, age, and marital status.

	Pre-Plague, 1740–1741	Plague, 1742–1743	Post-Plague, 1744–1745
Gender, female %	47.3%	48.1%	44.4%
Age, adult (≥18) %	62.2%	76.8%	65.0%
Marital status, single %	43.6%	23.6%	33.5%

**Table 3:** Ethnic distribution of mortality in Cordova and Santa Fe.

Ethnicity	Pre-Plague, 1740–1741	Plague, 1742–1743	Post-Plague, 1744–1745
Spanish	14.8%	13.6%	17.4%
Creole	42.6%	52.5%	46.2%
Indigenous/African	30.3%	27.1%	18.9%
*Pardo*	5.7%	2.3%	3.8%
Mulatto	2.5%	1.7%	0.8%
Mestizo	8.2%	4.5%	3.0%
Total	100%	100%	100%

Spanish: purely European descent; Creole: European descent born in the Americas; Indigenous/African: combined due to historical record practices not distinctly maintaining separate records for each group; *pardo*: mixed European, African, and sometimes Indigenous descent; mulatto: mixed European and African descent; mestizo: mixed Indigenous and European descent.

## Data Availability

Data are contained within the article.
